# Supplementary material for: The assembly of the Mitochondrial Complex I Assembly complex uncovers a redox pathway coordination
Source: Nat Commun. 2023 Dec 12;14:8248. doi: 10.1038/s41467-023-43865-0 (PMC10716376; doi:10.1038/s41467-023-43865-0)
Supplement: Supplementary file 1 — Supplementary Information [file 41467_2023_43865_MOESM1_ESM.pdf]

The assembly of the Mitochondrial Complex I Assembly complex uncovers a redox pathway coordination

Lindsay McGregor, Samira Acajjaoui, Ambroise Desfosses, Melissa Saïdi, Maria Bacia-Verloop, Jennifer J. Schwarz, Pauline Juyoux, Jill von Velsen, Matthew W. Bowler, Andrew A. McCarthy, Eaazhisai Kandiah, Irina Gutsche*, & Montserrat Soler-Lopez*

SUPPLEMENTAL INFORMATION

SUPPLEMENTARY FIGURES

Supplementary Figure 1. Biophysical characterisation of the $ACAD9_{WT}$-$ECSIT_{CTER}$ complex.

Supplementary Figure 2. Affinity analysis of the $ACAD9_{WT}$-$ECSIT_{CTER}$ complex.

Supplementary Figure 3. Cryo-EM processing strategies.

Supplementary Figure 4. Model to map fits of $ACAD9_{WT}$-$ECSIT_{CTER}$, $ACAD9_{WT}$ and $ACAD9_{S191A}$.

Supplementary Figure 5. Data quality assessment of the $ACAD9_{WT}$-$ECSIT_{CTER}$, $ACAD9_{WT}$ and $ACAD9_{S191A}$ datasets.

Supplementary Figure 6. AlphaFold2 (AF2) calculations predict the same ECSIT binding as that observed in our experimental model.

Supplementary Figure 7. Cryo-EM structure of ACAD9 unbound.

Supplementary Figure 8. Analysis of bonding interactions within ACAD9-$ECSIT_{CTER}$, with comparison to ACAD9 alone and VLCAD.

Supplementary Figure 9. Quaternary structure of VLCAD reveals insights into the bottom loop residues and the accessibility to the ECSIT binding site in ACAD9.

Supplementary Figure 10. Phosphorylation of ECSIT perturbs ACAD9-ECSIT subcomplex formation.

SUPPLEMENTARY TABLES

Supplementary Table 1. Cryo-EM data collection, processing and model refinement statistics for $ACAD9_{WT}$-$ECSIT_{CTER,}$ $ACAD9_{WT}$ and $ACAD9_{S191A}$.

Supplementary Table 2. Summary of all ACAD9-$ECSIT_{CTER}$ mutants designed to probe the effects of interaction site residues on complex formation.

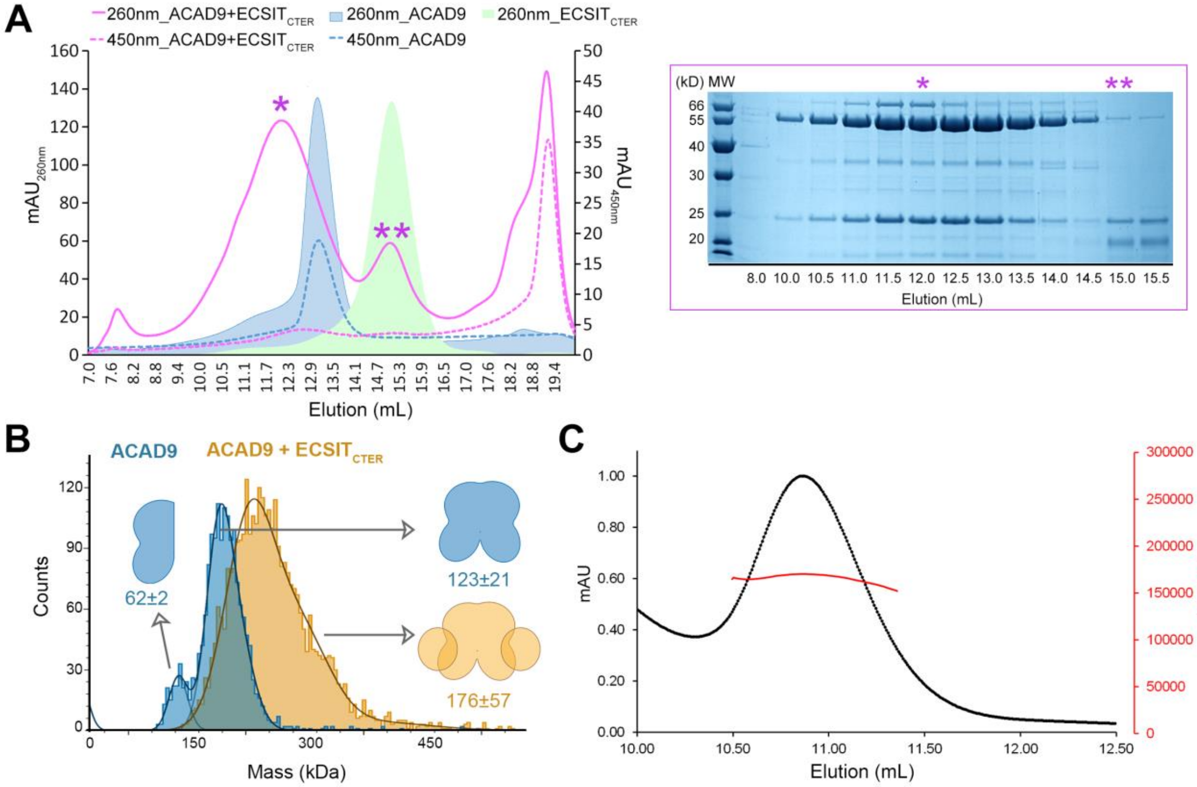

Supplementary Figure 1: Biophysical characterisation of the ACAD9$_{WT}$-ECSIT$_{CTER}$ complex. (A) Left, SEC elution profiles of ACAD9$_{WT}$ and ECSIT$_{CTER}$ alone and in complex monitored using UV wavelengths of 260 nm and 450 nm. ACAD9$_{WT}$ elutes at ~13 mL (filled blue peak) with FAD bound (dotted blue line); ECSIT$_{CTER}$ elutes ~15.3 mL (filled green peak). The ACAD9$_{WT}$-ECSIT$_{CTER}$ complex elutes ~12mL as indicated by a * on the 260 nm signal (solid pink line). The corresponding 450 nm signal (dotted pink line) indicates there is no FAD present in the ACAD9$_{WT}$-ECSIT$_{CTER}$ complex and instead is eluted much later as an unbound FAD molecule ~19 mL. Right, SDS-PAGE gel showing the eluted fractions corresponding to the ACAD9$_{WT}$-ECSIT$_{CTER}$ complex. The * corresponds to the apex of the ACAD9$_{WT}$-ECSIT$_{CTER}$ elution peak as shown in the chromatogram. **(B)** Mass photometer analysis shows the primary species in the ACAD9$_{WT}$ sample (blue) is the ACAD9$_{WT}$ dimer with a MW of ~123±21 kDa, with an additional smaller peak corresponding to the ACAD9$_{WT}$ monomer species with a MW of 62±2 kDa in much lower proportion. A shift in MW is seen in the formation of the ACAD9$_{WT}$-ECSIT$_{CTER}$ complex, resulting in an estimated MW of 176±57, corresponding to a dimer of ACAD9$_{WT}$ and two ECSIT$_{CTER}$ monomers, as described in the schematic (orange). **(C)** Analysis of the ACAD9$_{WT}$-ECSIT$_{CTER}$ complex by SEC coupled with multi-atomic light scattering (MALS). The elution profile was monitored by excess refractive index (left axis, red). The estimated atomic mass of the complex is given by the black line, indicating that the MW of the ACAD9$_{WT}$-ECSIT$_{CTER}$ complex is 170 kDa, measured on the right axis. Source data and uncropped SDS-PAGE gel are provided as a Source Data file.

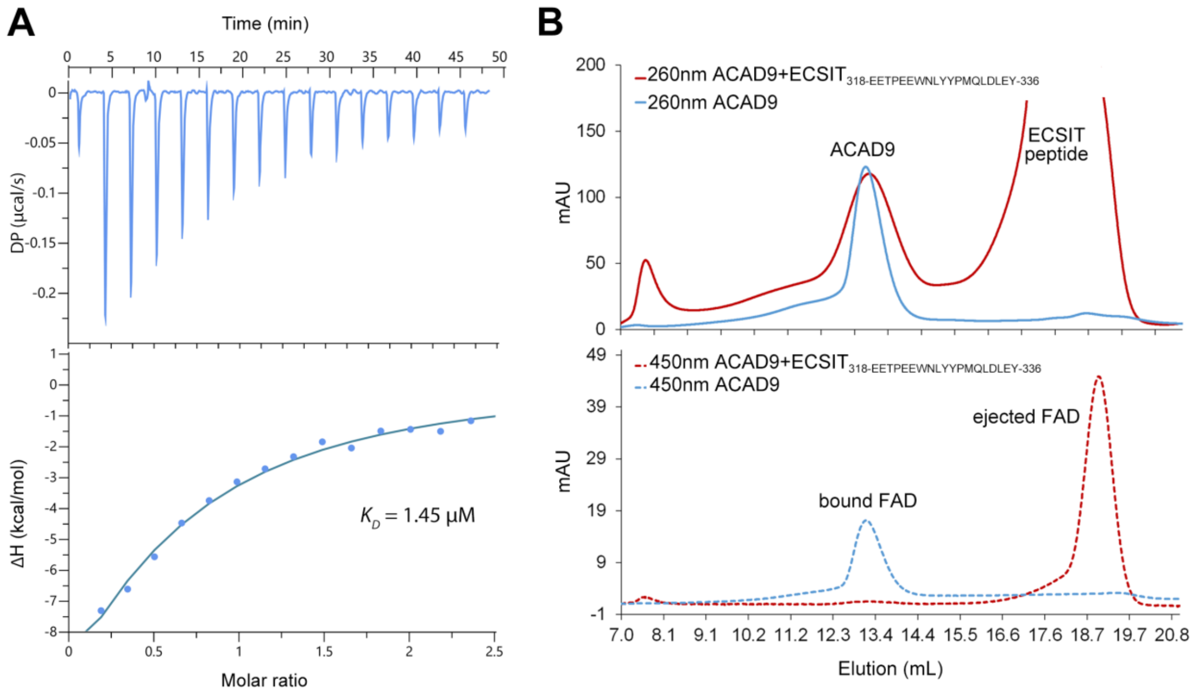

Supplementary Figure 2: Affinity analysis of the $ACAD9_{WT}$-$ECSIT_{CTER}$ complex. (A) ITC binding assay for the binding affinity between $ACAD9_{WT}$ and $ECSIT_{CTER}$. The equilibrium dissociation constant (K_D) of the $ACAD9_{WT}$-$ECSIT_{CTER}$ complex is 1.45 µM. The experiment was repeated thrice with similar results. **(B)** SEC elution profiles of $ACAD9_{WT}$ alone and reconstituted with the ECSIT peptide (residues 318-336), monitored using UV wavelengths of 260 nm and 450 nm. The SEC elution profile for $ACAD9_{WT}$ (blue) shows the elution around 13 mL (top panel) with the corresponding FAD signal eluting at the same volume (blue, bottom panel), indicating FAD-bound $ACAD9_{WT}$. When reconstituted with the ECSIT peptide, the elution profile (red, top panel) shows no significant shift from the $ACAD9_{WT}$ alone profile (blue) however there is clearly a loss of FAD from the $ACAD9_{WT}$ dimer (red, bottom panel), induced by the ECSIT peptide. Source data are provided as a Source Data file.

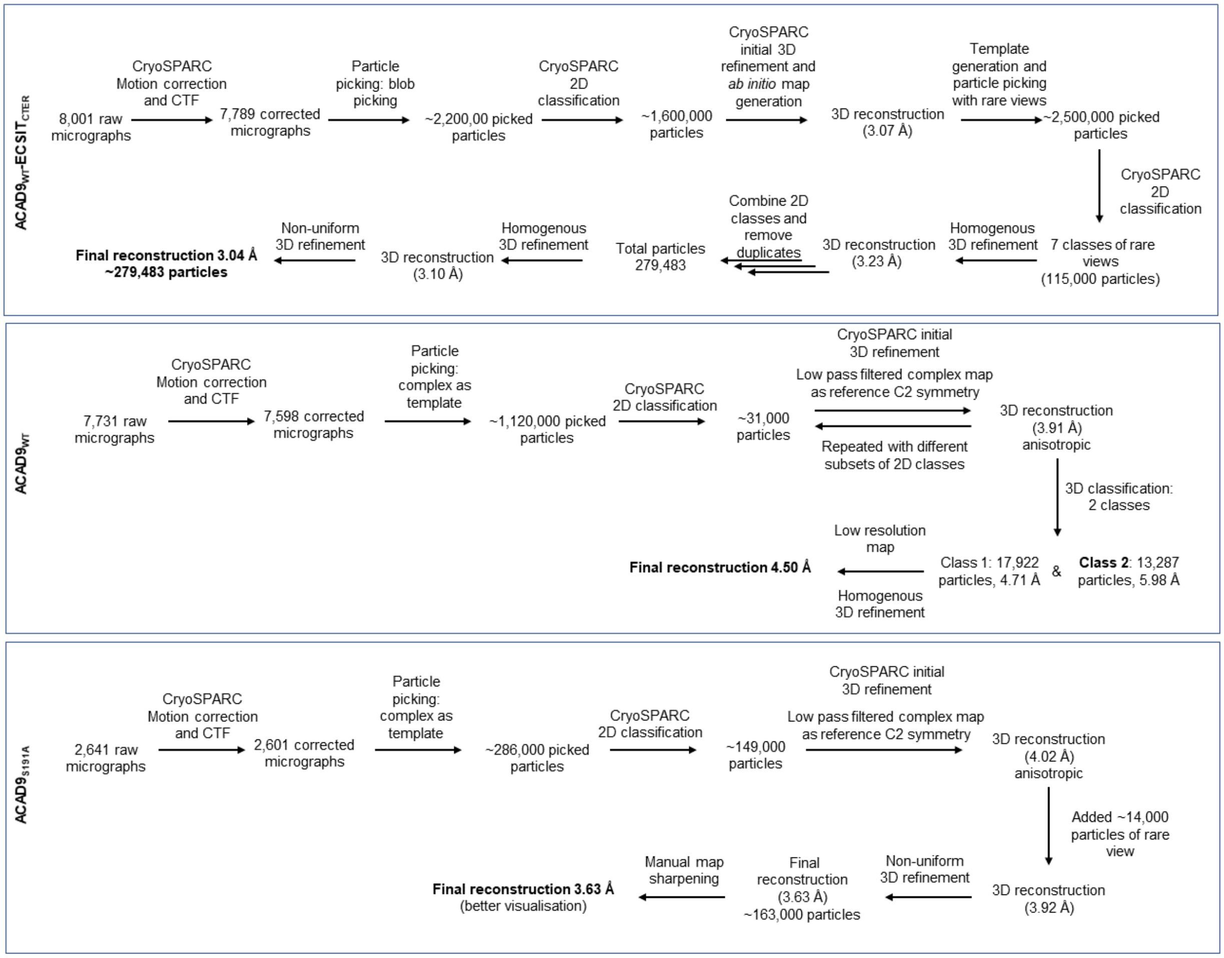

Supplementary Figure 3: Cryo-EM processing strategies. Imaging processing pipeline for all datasets, describing the software packages used and details of the decision-making process at each step. Representative micrographs showing individual particles for each dataset are provided in Supplementary Fig. 5A.

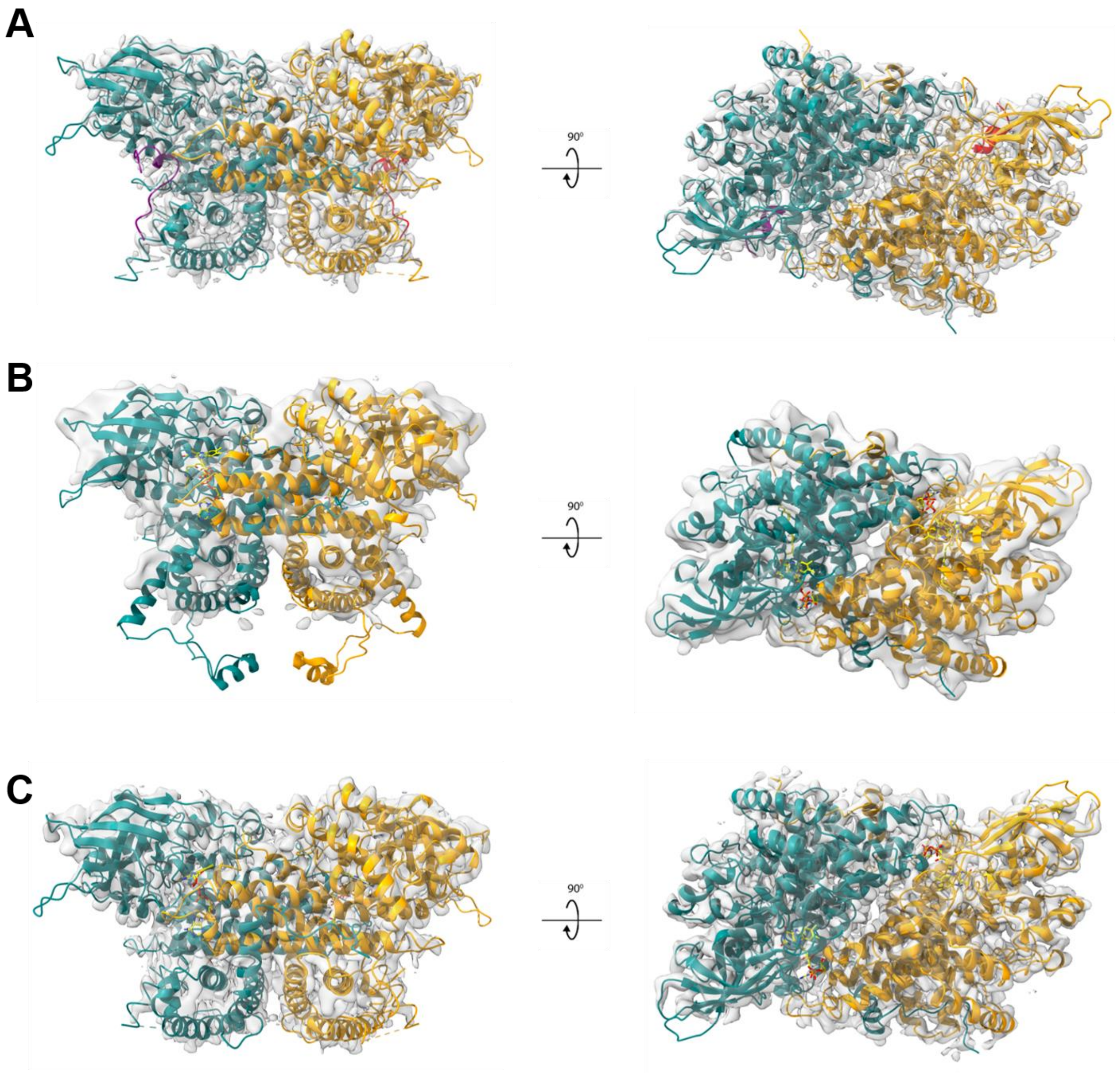

Supplementary Figure 4: Model to map fits of ACAD9$_{WT}$-ECSIT$_{CTER}$, ACAD9$_{WT}$ and ACAD9$_{S191A}$. (**A**) Two views of the final model of ACAD9$_{WT}$-ECSIT$_{CTER}$ (PDB-8PHE) with the final map of the complex (EMD-17659). (**B**) Two views of the ACAD9 AF2 model (res. 38-621) fitted into the final map of ACAD9$_{WT}$ (EMD-17661). (**C**) Two views of the final model of ACAD9$_{S191A}$ (PDB-8PHF) with the final map of ACAD9$_{S191A}$ (EMD-17660). The contour levels in ChimeraX used to generate these views are 0.75 (**A**), 0.078 (**B**), and 0.55 (**C**). The two ACAD9 monomers are shown in teal and light orange, respectively. ECSIT$_{CTER}$ segments are in purple and red. The FAD cofactor is shown as a yellow atomic stick model.

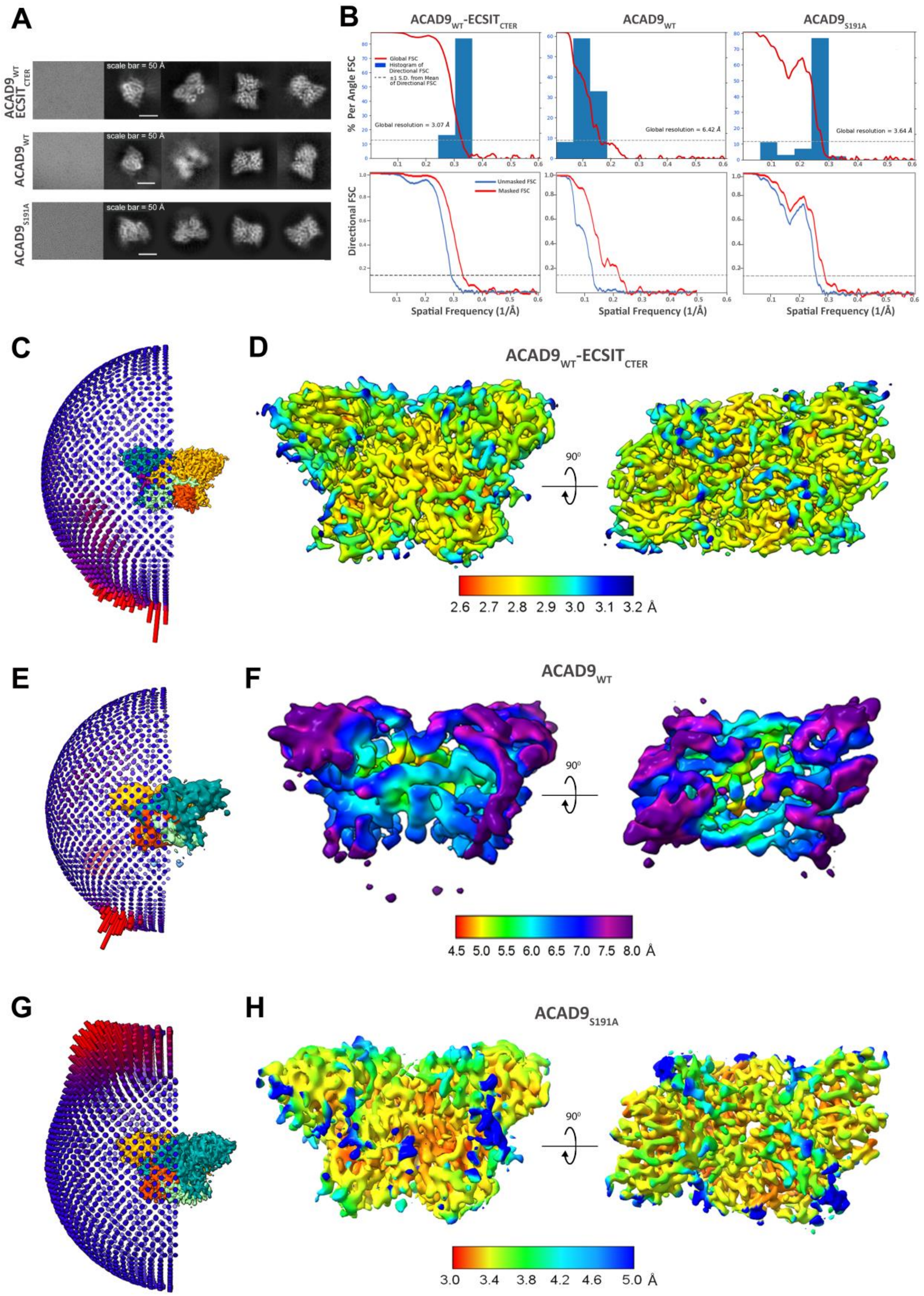
A
B
ACAD9WT-ECSITCTER
ACAD9WT
ACAD9S191A
scale bar = 50 Å
% Per Angle FSC
Global FSC
Histogram of Directional FSC
±1 S.D. from Mean of Directional FSC
Global resolution = 3.07 Å
Global resolution = 6.42 Å
Global resolution = 3.64 Å
Directional FSC
Unmasked FSC
Masked FSC
Spatial Frequency (1/Å)
C
D
ACAD9WT-ECSITCTER
90°
2.6 2.7 2.8 2.9 3.0 3.1 3.2 Å
E
F
ACAD9WT
4.5 5.0 5.5 6.0 6.5 7.0 7.5 8.0 Å
G
H
ACAD9S191A
3.0 3.4 3.8 4.2 4.6 5.0 Å

Supplementary Figure 5: Data quality assessment of the $ACAD9_{WT}$-$ECSIT_{CTER}$, $ACAD9_{WT}$ and $ACAD9_{S191A}$ datasets. (A) Thumbnails of typical micrographs representative of the datasets and selected 2D classes for $ACAD9_{WT}$-$ECSIT_{CTER}$ (top), $ACAD9_{WT}$ (centre) and $ACAD9_{S191A}$ (bottom). **(B)** Histograms of the directional FSC (top), plotting the global half-map FSC (solid red line) and where the blue bars are a histogram of 100 such values sampled evenly over the 3D FSC, indicating the global resolution for $ACAD9_{WT}$-$ECSIT_{CTER}$ (left), $ACAD9_{WT}$ (centre) and $ACAD9_{S191A}$ (right) respectively. The Gold-standard Fourier Shell Correlation (FSC) curves (bottom) for both the unmasked (blue) and masked (red) maps for $ACAD9_{WT}$-$ECSIT_{CTER}$ (left), $ACAD9_{WT}$ (centre) and $ACAD9_{S191A}$ (right) respectively. The 0.143 cut-off is represented by a grey dotted line. **(C)** Angular distribution of the particles used in the final reconstruction with the final ACAD9-$ECSIT_{CTER}$ map for reference **(D)** Local resolution distribution of the final map as calculated in CryoSPARC, showing front and top particle views. **(E)** Angular distribution of the particles used in the final reconstruction with the final $ACAD9_{WT}$ map for reference. **(F)** Local resolution distribution of the final map as calculated in CryoSPARC, showing front and top particle views. The local resolution is higher in the core of the protein (~4.0 Å) whereas the solvent accessible areas of the protein are less well resolved (~8.0 Å). **(G)** Angular distribution of the particles used in the final reconstruction with the final $ACAD9_{S191A}$ map for reference. **(H)** Local resolution distribution of the final map as calculated in CryoSPARC, showing front and top particle views.

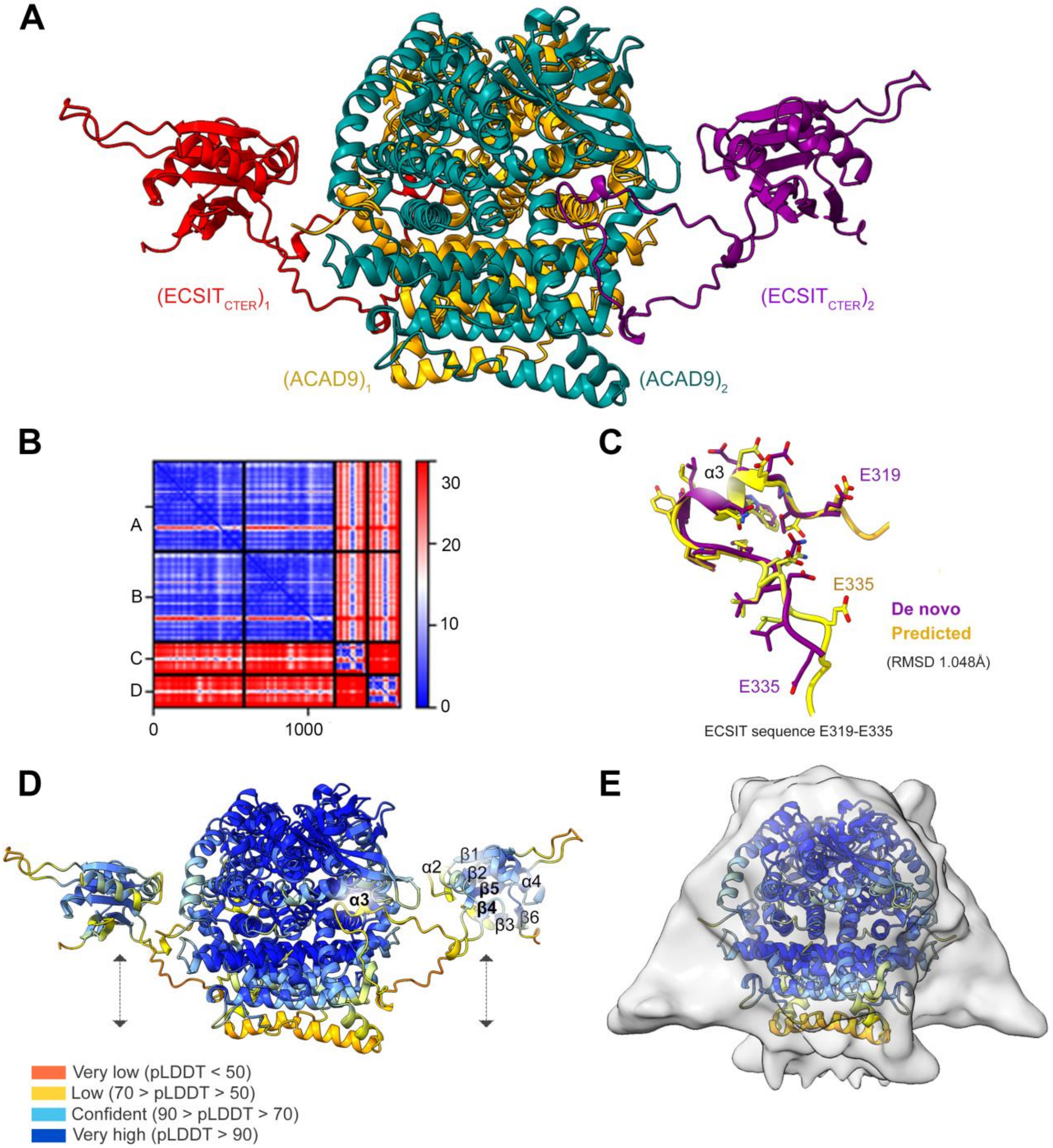

Supplementary Figure 6: AlphaFold2 (AF2) calculations predict the same ECSIT binding as that observed in our experimental model. (A) Calculated model from AF2 showing the dimer of ACAD9 and two ECSIT$_{CTER}$ monomers from a side view. The ACAD9 monomers are shown in teal and orange, whereas the two ECSIT monomers are shown in purple and red, one bound to each monomer of ACAD9. **(B)** The Predicted Aligned Error (PAE) plot shown indicates a low level of error in this docked model, indicating that while the prediction has a low per-residue confidence metric in the orientation of the β4-β5 loop (containing the 3_{10}-helix, named α3) that binds to ACAD9, the low PAE error suggests that the prediction is confident of their relative positions within the subcomplex. The y-axis indicates A and B correspond to the first and

second ACAD9 monomers and C and D correspond to the two ECSIT monomers. The x-axis gives the residue number from A -> D across the whole complex. The PAE scores between 0 (blue, low error) and 30 (red, high error). **(C)** An alignment of the ECSIT loop from the AF2 prediction (yellow) with our experimental model (purple) shows high agreement (RMSD = 1.048 Å). **(D)** The docked model is coloured according to the predicted local distance difference test (pLDDT) score, indicating a per-residue estimate of confidence. The colour of the ECSIT binding loop (residues 320-334 corresponding to the β4-α3-β5 loop in the AF2 model) are coloured yellow, indicating low per-residue confidence, however, the PAE in (**B**) indicates a low error score. **(E)** Although in our earlier low-resolution map of ACAD9-ECSIT$_{CTD}$ we could only dock the ACAD9 homodimer[11], at a low threshold it shows a density extension that appears to be very closed to the ECSIT loop flexibly connected to strands β4 and β5 of the RNAseH-like domain, suggesting that this domain could be positioned within the extra density with only a minor adjustment of the long predicted β4-α3-β5 loop, as proposed by the grey arrows in **(D)**. Residues with a pLDDT score < 30 are omitted for clarity.

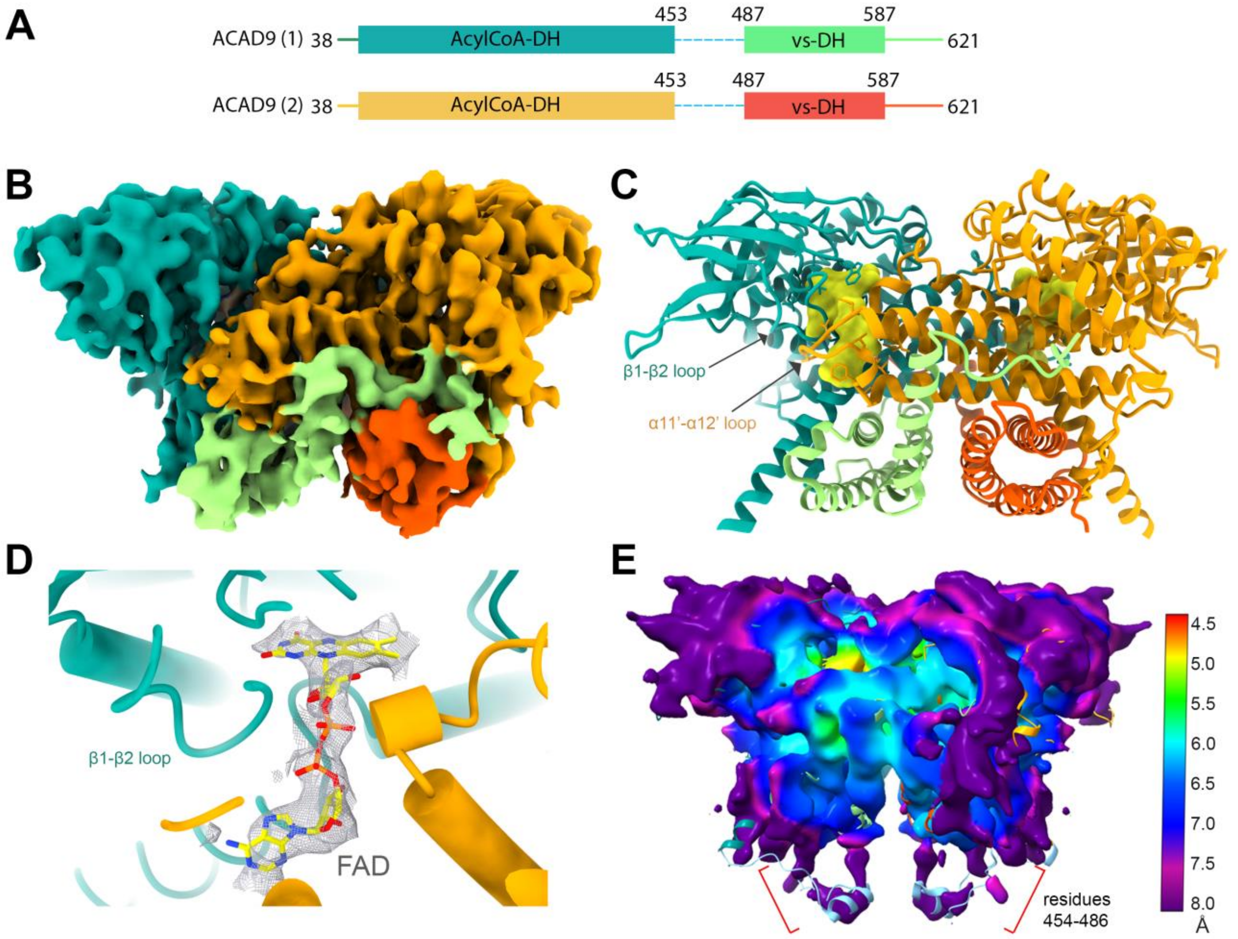

Supplementary Figure 7: Cryo-EM structure of ACAD9 unbound. (A) Schematic of the sequence of ACAD9 modelled in the cryo-EM map. The two ACAD9 monomers are labelled (1) and (2), with their dehydrogenase domains shown in teal and light orange, and their vestigial domains in light green and dark orange, respectively. The dashed lines indicate ACAD9 residues 454-486 absent from the final model. **(B)** Cryo-EM map of ACAD9$_{S191A}$ viewed from the front, coloured as in **Fig. 1**. **(C)** Final model of ACAD9$_{S191A}$ with the density of the FAD highlighted in yellow. **(D)** Close-up of the FAD density in the ACAD9$_{S191A}$ cofactor pocket, confirming that ACAD9$_{S191A}$ is in the active Acyl-CoA dehydrogenase form of ACAD9. **(E)** The final reconstruction of ACAD9$_{WT}$ depicted as a local resolution map. Reducing the threshold enables visualization of faint densities at the base of the protein, close to the location of the unmodelled residues 454-486. The homology model was modified to fit these residues (light blue) into the low-resolution density, showing that these previously unmodelled residues position themselves close to the base of the ACAD9 dimer.

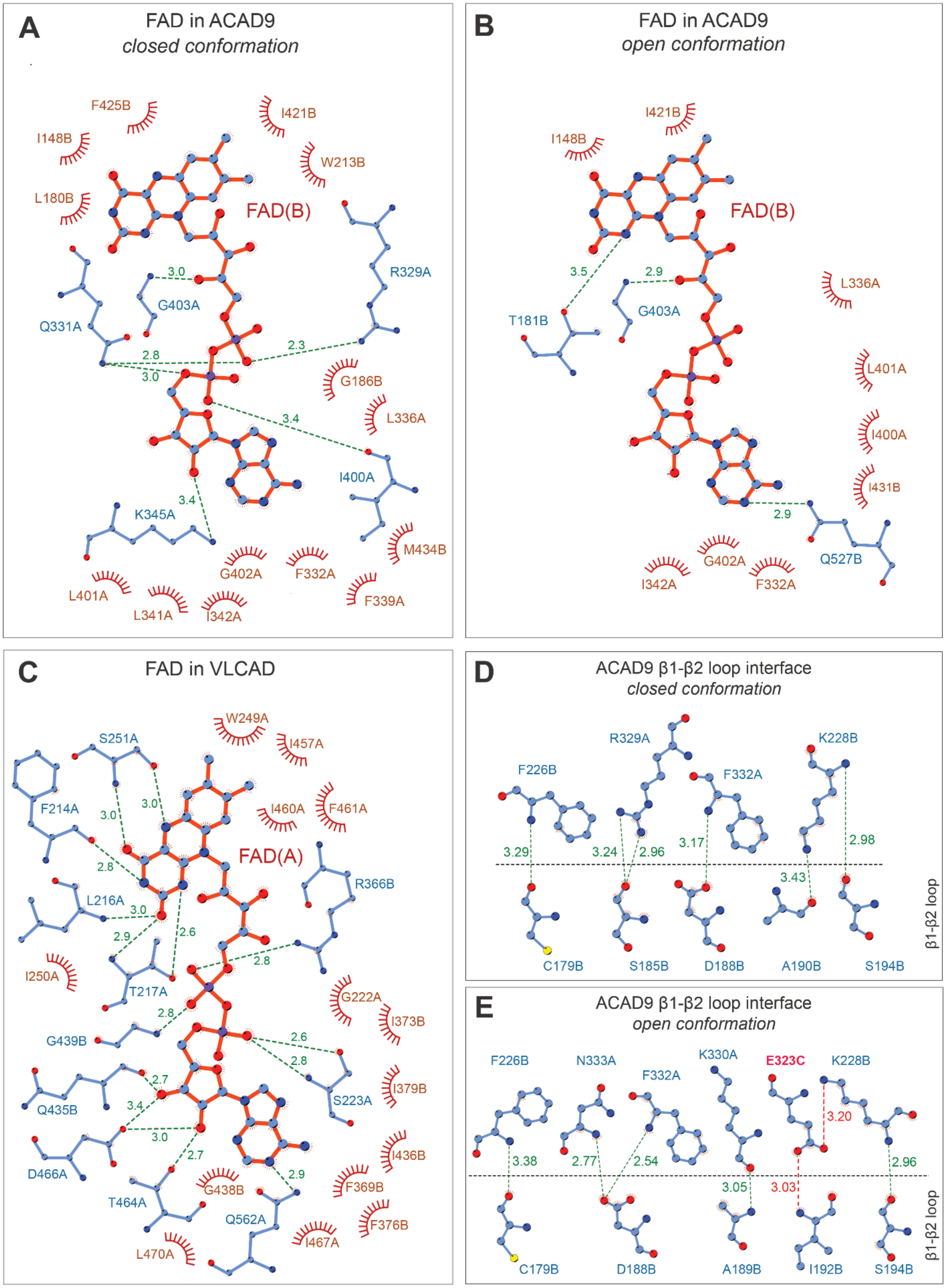

A
FAD in ACAD9
closed conformation
F425B
I421B
I148B
W213B
L180B
FAD(B)
R329A
3.0
G403A
Q331A
2.3
2.8
3.0
G186B
3.4
L336A
I400A
3.4
K345A
M434B
G402A
F332A
L401A
F339A
L341A
I342A
B
FAD in ACAD9
open conformation
I421B
I148B
FAD(B)
3.5
2.9
L336A
T181B
G403A
L401A
I400A
I431B
2.9
Q527B
G402A
I342A
F332A
C
FAD in VLCAD
W249A
S251A
I457A
3.0
I460A
F461A
F214A
3.0
2.8
FAD(A)
R366B
L216A
3.0
2.9
2.6
I250A
2.8
T217A
G222A
2.8
I373B
G439B
2.6
2.8
2.7
I379B
S223A
Q435B
3.4
3.0
I436B
D466A
2.7
2.9
G438B
F369B
T464A
Q562A
F376B
I467A
L470A
D
ACAD9 β1-β2 loop interface
closed conformation
R329A
K228B
F226B
F332A
3.29
3.24
2.96
3.17
2.98
3.43
β1-β2 loop
C179B
S185B
D188B
A190B
S194B
E
ACAD9 β1-β2 loop interface
open conformation
F226B
N333A
K330A
E323C
K228B
F332A
3.20
3.38
2.77
2.54
2.96
3.05
3.03
β1-β2 loop
C179B
D188B
A189B
I192B
S194B

Supplementary Figure 8: Analysis of the FAD binding pocket within ACAD9-ECSIT$_{CTER}$, with comparison to ACAD9 alone and VLCAD. (**A**) LIGPLOT diagrams showing the details of the FAD bonding interactions present in the closed conformation of ACAD9. Several hydrogen bonds between the neighbouring protomer of the protein and the phosphate groups of the FAD retain the cofactor within ACAD9. Additionally, a number of hydrophobic residues also surround the FAD molecule in the closed conformation, forming and stabilising the FAD pocket. (**B**) In contrast, the number of bonding interactions between the FAD coordinates (taken from ACAD9$_{S191A}$ and inserted into the deflavinated ACAD9-ECSIT$_{S191A}$ structure, open conformation) and the surrounding protein is vastly reduced. (**C**) The increased number of interactions between VLCAD and FAD in comparison to ACAD9 is clear. The flipped orientation of the isoalloxazine ring also permits additional hydrogen bonds, contributing to the stability of the cofactor. A network of hydrophobic residues surrounds the cofactor forming the FAD pocket, similar to that observed in ACAD9. (**D**) DIMPLOT diagrams showing a closer analysis of the β1-β2 loop bonding interactions within ACAD9 between closed and open **(E)** conformations reveals the change in residues responsible for stabilising the loop in each conformational state. Residues of the β1-β2 loop are displayed at the bottom side of the interface while the interacting residues are represented above. The ECSIT Glu323 residue (in red) interacts with ACAD9 I192N and K228NZ (in red), replacing the ACAD9 A190O-K228NZ hydrogen bond in the closed conformation. Relevant hydrogen bonds are displayed as green lines with the distances given in Å and surrounding hydrophobic residues are displayed with red half-moons.

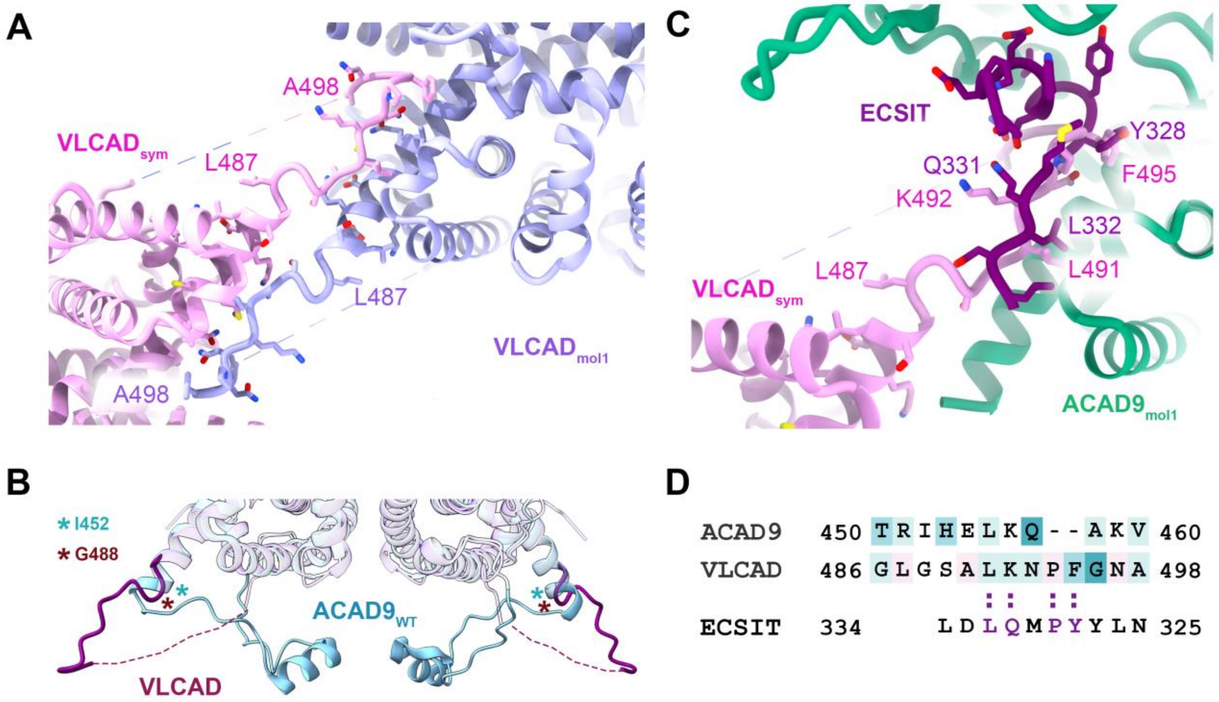

Supplementary Figure 9: Quaternary structure of VLCAD reveals insights into the bottom loop residues and the accessibility to the ECSIT binding site in ACAD9. (A) The crystal packing of VLCAD is formed mainly by symmetric contacts involving the 485-498 loops of symmetry- related molecules. Closeup of the interaction between symmetry-related loops. **(B)** The structural comparison of the ACAD9$_{WT}$ 454-486 residues with the VLCAD 490-522 residues reveal that that flexible region adopts an extended conformation in VLCAD as compared to the closed conformation observed in ACAD9$_{WT}$ (Supplementary Fig. 7E). **(C)** Structural alignment of the VLCAD quaternary structure and the ACAD9-ECSIT$_{CTER}$ structure, highlighting those residues occupying similar positions in each respective model. **(D)** Pairwise sequence alignment of ACAD9 and VLCAD around the 35-residue region that has the greatest difference between the ACAD9 and VLCAD and that has been postulated as the determining feature for CI assembly. Conserved residues between ACAD9 and VLCAD are highlighted in purple. The residues are coloured based on the ConSurf prediction (Fig. 4B), highlighting the poor conservation within ACAD9 and VLCAD in this region. Structural alignment of the VLCAD and ACDA9-ECSIT$_{CTER}$ structures shows that the VLCAD 484-498 loop aligns well with the ECSIT sequence visible in the cryo-EM structure, suggesting that the VLCAD loop actually occupies, and perhaps blocks, the ECSIT binding site, in contrast to ACAD9.

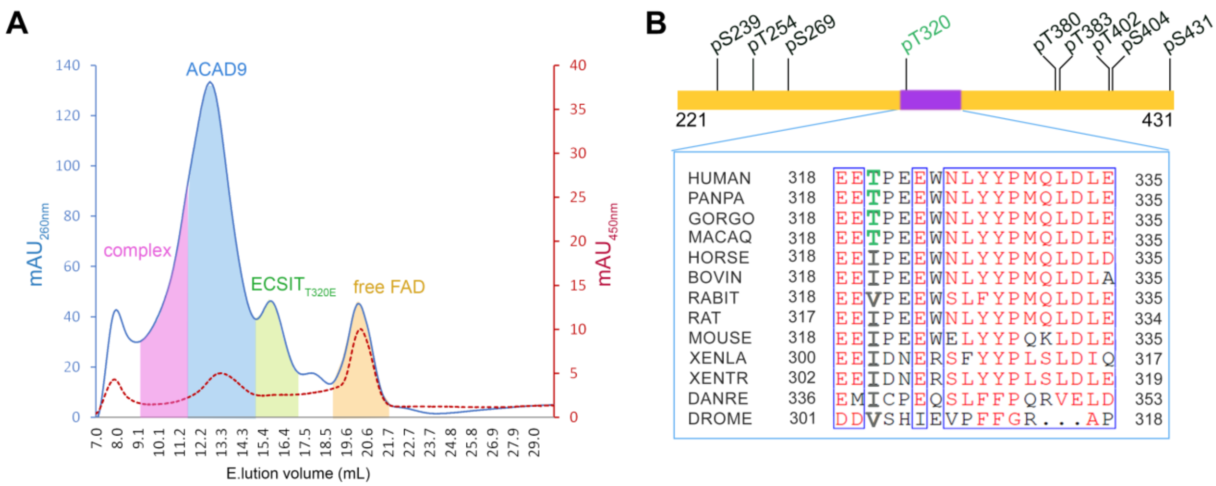

Supplementary Figure 10: Phosphorylation of ECSIT perturbs ACAD9-ECSIT subcomplex formation. (A) SEC profile of ACAD9$_{WT}$-ECSIT$_{T320E}$ phosphomimetic. In comparison to ACAD9$_{WT}$-ECSIT$_{CTER}$ (Supplementary Fig. 1A), the major species present after subcomplex reconstitution is FAD-bound ACAD9$_{WT}$ alone (filled blue peak, dotted red line). There is no clear peak that can be attributed to the ACAD9$_{WT}$-ECSIT$_{CTER}$ subcomplex, instead a shoulder indicates the formation of subcomplex aggregates (filled pink peak), accounting for the free FAD (dotted red line). The loss of subcomplex formation is consistent with results seen in Mass Photometry experiments (Fig. 6D). **(B)** Diagram summarising the phosphorylation sites of ECSIT$_{CTER}$ identified by mass spectrometry. The sequence observed in the cryo-EM map is highlighted in purple and the potential Thr320 phosphorylation site in green. Multiple sequence alignment of this region between different species reveals a high conservation. However, Thr320 seems to have appeared in higher primates as a replacement of a hydrophobic residue, suggesting that this substitution might have appeared as an evolutionary shift in function.

Supplementary Table 1. Cryo-EM data collection, processing and model refinement statistics for $ACAD9_{WT}$-$ECSIT_{CTER}$, $ACAD9_{WT}$ and $ACAD9_{S191A}$.

	**$ACAD9_{WT}$-$ECSIT_{CTER}$**	**$ACAD9_{WT}$**	**$ACAD9_{S191A}$**
EMD ID	17659	17660	17661
PDB ID	8PHE	--	8PHF
Data collection and processing			
Magnification	165k	165k	105k
Voltage (kV)	300	300	300
Electron exposure (e^-/Å^2)	41.67	40.20	62.50
Defocus range (uM)	0.7 – 2.5	0.7 – 2.5	0.8 – 2.2
Pixel size (Å)	0.827	0.827	0.840
Symmetry imposed	C2	C2	C2
Initial particle images (no.)	2 214 363	1 120 000	286 397
Final particle images (no.)	279 483	13 287	163 409
Map resolution (Å)	3.04	5.98	3.63
FSC threshold	0.143	0.143	0.143
Map resolution range (Å)	2.6 – 3.2	5.0 – 8.0	3.0 – 5.0
Fitting/Refinement			
Used Software	Chimera, Phenix	Chimera, Phenix	Chimera, Phenix
Initial model used	VLCAD-based homology	ACAD9 AF2 model & $ACAD9_{WT}$-$ECSIT_{CTER}$	$ACAD9_{WT}$-$ECSIT_{CTER}$
Map resolution (Å)	3.04	--	3.63
FSC threshold	0.5		0.5
Map sharpening B factor (Å^2)	-171	--	-180
Model composition			
Non-hydrogen atoms	8951		8651
Protein residues	1136	--	1095
Ligands (ADP)	0		2
Waters	38		0
B factors (Å^2)			
Protein	44.92	--	23.09
Ligand	0.00		32.58
Waters	41.35		--
R.m.s. deviations			
Bond lengths (Å)	0.003	--	0.004
Bond angles (°)	0.512		0.614
Validation			
MolProbity score	1.75	--	1.90
Clashscore	8.78		11.53
Poor rotamers (%)	0.00		0.11
Ramachandran plot			
Favored (%)	95.91	--	95.40
Allowed (%)	4.09		4.60
Disallowed (%)	0.00		0.00

Supplementary Table 2. Summary of ACAD9-$ECSIT_{CTER}$ mutants designed to probe the effects of interaction site residues on complex formation.

Complex mutant (in bold)	Rationale	Mass Photometry (MP)	Dynamic Light Scattering (DLS)	Conclusion
$ACAD9_{WT}$:$ECSIT_{CTER}$-**E323A**	Formation of salt bridge between ECSIT Glu323 and ACAD9 Lys228. Removal of sidechain and salt bridge capabilities of ECSIT Glu323.	Complex formation significantly disrupted, MW of the major species similar to that of unbound ACAD9.	Major species present has MW similar to ACAD9 dimer.	Loss of ECSIT Glu323 salt bridge interaction detrimental to complex formation.
$ACAD9_{WT}$: $ECSIT_{CTER}$-**W324A**	Disruption of hydrogen bond between -OH of ACAD9 Ser191 and -NH of ECSIT Trp324. Removal of aromatic sidechain and hydrogen bonding -NH of ECSIT Trp324.	Complex formation significantly disrupted, MW of the major species similar to that of unbound ACAD9.	Major species present has MW similar to ACAD9 dimer.	Loss of ECSIT Trp324 aromatic system and hydrogen bonding interaction detrimental to complex formation.
$ACAD9_{WT}$:$ECSIT_{CTER}$-**Y327F**	Removal of the -OH of ECSIT Tyr327 that points into the ACAD9-ECSIT binding site with no obvious hydrogen bonding partner.	Formation of the complex observed, disruption of the complex stability.	Estimated MW higher than MW of ACAD9 dimer, however, large error present due to the contribution from the additional species observed in the MP data.	Removal of ECSIT Tyr327 hydroxyl group affects complex formation/stability but does not prevent interaction.
$ACAD9_{WT}$:$ECSIT_{CTER}$-**Y327A**	Removal of aromatic sidechain and -OH of ECSIT Tyr327 that points into the ACAD9-ECSIT binding site with no obvious hydrogen bonding or hydrophobic partners.	Complex formation significantly disrupted, MW of the major species similar to that of unbound ACAD9.	Major species present has MW similar to ACAD9 dimer.	Loss of ECSIT Tyr327 aromatic system and hydrogen bonding interaction detrimental to complex formation.
$ACAD9_{WT}$:$ECSIT_{CTER}$-**Y328F**	Removal of the -NH of ECSIT Tyr328 that points into the ACAD9-ECSIT binding site with no obvious hydrogen bonding partner.	Complex formation significantly disrupted, MW of main species similar to that of unbound ACAD9.	Major species present has MW similar to ACAD9 dimer.	Removal of ECSIT Tyr327 hydroxyl group detrimental to complex formation.
$ACAD9_{WT}$:$ECSIT_{CTER}$-**Y328A**	Removal of aromatic sidechain and -NH of ECSIT Tyr328 that points into the ACAD9-ECSIT binding site with no obvious hydrogen bonding partner.	Formation of the complex observed, minor disruption of the complex stability.	Major species present has MW similar to ACAD9 dimer, however, large error present due to the contribution from the additional species observed in the MP data.	Loss of ECSIT Tyr328 aromatic system and hydrogen bonding interaction perturbs stability of complex formation.
ACAD9-**S191A**:$ECSIT_{CTER}$	Disruption of hydrogen bond between -OH of ACAD9-Ser191 and -NH of ECSIT Trp324. Removal of the ACAD9 Ser191 hydroxyl group.	Formation of the complex observed, disruption of the complex stability.	Much higher MW species than expected for complex formation.	$ACAD9_{S191A}$ appears to have a higher stability than $ACAD9_{WT}$, however, disruption of a hydrogen bond in active site perturbs complex formation with $ECSIT_{CTER}$.

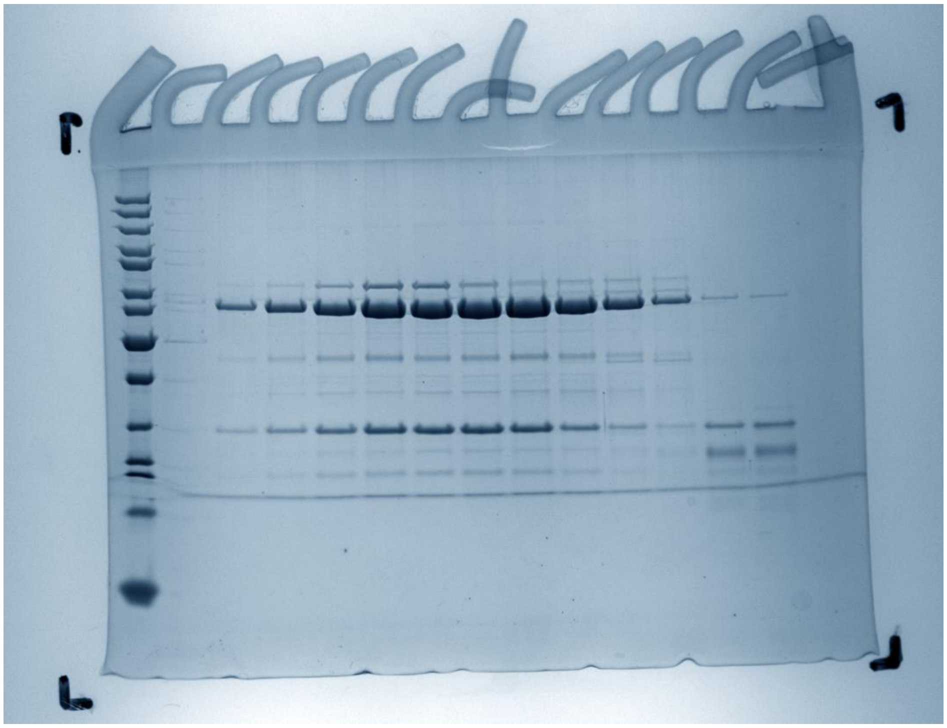

Uncropped SDS-PAGE gel from Supplementary Figure 1A.
