# Supplementary material for: The assembly of the Mitochondrial Complex I Assembly complex uncovers a redox pathway coordination
Source: Nat Commun. 2023 Dec 12;14:8248. doi: 10.1038/s41467-023-43865-0 (PMC10716376; doi:10.1038/s41467-023-43865-0)
Supplement: Supplementary file 3 — Description of Additional Supplementary Files [file 41467_2023_43865_MOESM3_ESM.pdf]

Description of Additional Supplementary Files

File Name: Supplementary Data 1
Description: Details of ACAD9, ECSIT and VLCAD primers and plasmids used in experiments (all variants).

File Name: Supplementary Data 2
Description: Phosphosites detected by mass spectrometry data from *in vitro* $ECSIT_{CTER}$ phosphorylation assays.

File Name: Supplementary Data 3
Description: Phosphosites detected by mass spectrometry data from *ex cellulo* $ECSIT_{CTER}$ phosphorylation assays.

File Name: Supplementary Movie 1
Description: **Close up of ACAD9WT--$ECSIT_{CTER}$ binding site with the $ACAD9_{-S191A}$ FAD cofactor superimposed.** The $ECSIT_{CTER}$ residues modelled in the cryo-EM map and their proximity to the ACAD9WT flexible β1-β2 loop are shown as sticks (bold purple and teal respectively). In addition, the FAD cofactor coordinates taken from the $ACAD9_{--S191A-}$- structure are shown. This helps to visualise how the β1-β2 loop of ACAD9 plays a gatekeeper role, opening upon ECSIT binding and allowing the insertion of its 3_{10-}helix. Removal of this barrier is thought to destabilise the FAD cofactor environment, leading to deflavination of ACAD9. This can be compared directly with Supplementary Video 2.

File Name: Supplementary Movie 2
Description: **Close up of the $ACAD9_{S191A}$β1-β2 loop and FAD cofactor with $ECSIT_{CTER}$ superimposed. The ACAD9** β1-β2 loop in its closed conformation providing a barrier between the FAD cofactor and the solvent, thus acting as a gatekeeper. The clash between the atoms of ECSIT--CTER and the ACAD9β1-β2 loop is clearly shown, demonstrating that the ACAD9 β1-β2 loop cannot exist in the closed conformation while ECSIT is bound. The closed nature of this loop may contribute to the stability of the FAD cofactor environment, preventing deflavination in the absence of ECSIT. This can be directly compared with Supplementary Video 1.
