# Supplementary material for: The assembly of the Mitochondrial Complex I Assembly complex uncovers a redox pathway coordination
Source: Nat Commun. 2023 Dec 12;14:8248. doi: 10.1038/s41467-023-43865-0 (PMC10716376; doi:10.1038/s41467-023-43865-0)
Supplement: Supplementary file 9 — Reporting Summary [file 41467_2023_43865_MOESM9_ESM.pdf]

nature portfolio

Corresponding author(s): Montserrat Soler-Lopez, Irina Gutsche

Last updated by author(s): Nov 20, 2023

Reporting Summary

Nature Portfolio wishes to improve the reproducibility of the work that we publish. This form provides structure for consistency and transparency in reporting. For further information on Nature Portfolio policies, see our Editorial Policies and the Editorial Policy Checklist.

Statistics

For all statistical analyses, confirm that the following items are present in the figure legend, table legend, main text, or Methods section.

n/a	Confirmed	
☐	☒	The exact sample size (*n*) for each experimental group/condition, given as a discrete number and unit of measurement
☐	☒	A statement on whether measurements were taken from distinct samples or whether the same sample was measured repeatedly
☐	☒	The statistical test(s) used AND whether they are one- or two-sided<br>*Only common tests should be described solely by name; describe more complex techniques in the Methods section.*
☒	☐	A description of all covariates tested
☐	☒	A description of any assumptions or corrections, such as tests of normality and adjustment for multiple comparisons
☐	☒	A full description of the statistical parameters including central tendency (e.g. means) or other basic estimates (e.g. regression coefficient) AND variation (e.g. standard deviation) or associated estimates of uncertainty (e.g. confidence intervals)
☐	☒	For null hypothesis testing, the test statistic (e.g. *F*, *t*, *r*) with confidence intervals, effect sizes, degrees of freedom and *P* value noted<br>*Give P values as exact values whenever suitable.*
☒	☐	For Bayesian analysis, information on the choice of priors and Markov chain Monte Carlo settings
☒	☐	For hierarchical and complex designs, identification of the appropriate level for tests and full reporting of outcomes
☒	☐	Estimates of effect sizes (e.g. Cohen's *d*, Pearson's *r*), indicating how they were calculated

Our web collection on statistics for biologists contains articles on many of the points above.

Software and code

Policy information about availability of computer code

Data collection

Mass photometry data were collected using Refeyn AcquireMP 2.3.0 and Refeyn DiscoverMP 2.3.0 software packages.
Coomassie gels were collected using Image Lab Touch Software v2.3.0.07.
Cryo-EM data were screened using SerialEM 4.0 on a Thermofisher Glacios microscope and collected using the EPU package v2.10-v2.13 on a Thermofisher Titan Krios microscope.

Data analysis

ITC data analysis was carried out using the MicroCal PEAQ-ITC Analysis Software (Malvern Panalytical v1.41).
Mass photometry data analysis was done using Refeyn AcquireMP 2.3.0 and Refeyn DiscoverMP 2.3.0 software packages.
MALLS data analysis done with ASTRA software and visualised using OriginPro 9.0.
DLS data analysis with Zetasizer Software 8.01.4906. Cryo-EM image analysis done with cryoSPARC v3.2 software.
Model rigid body 3D reconstruction with ChimeraX v1.2.
Model refinement with PHENIX v1.18.2. Model visualisation with Coot v0.9.4.1.
Visualisation of model structures and scheme drawing of molecular interactions with PyMOL v4.60 and LigPlot+ v.2.2.8.
Predictive modelling using ColabFold v1.3.0 and AlphaFold Protein Structure Database AF2 v.2.2.4.
Mammalian cell data statistical analysis done with GraphPad Prism v.10 software.
Mass spectrometry data analysis done with MaxQuant v.1.6.17.0.
Gel imaging analysis with Image Lab Touch Software v2.3.0.07.

For manuscripts utilizing custom algorithms or software that are central to the research but not yet described in published literature, software must be made available to editors and reviewers. We strongly encourage code deposition in a community repository (e.g. GitHub). See the Nature Portfolio guidelines for submitting code & software for further information.

Data

Policy information about availability of data

All manuscripts must include a data availability statement. This statement should provide the following information, where applicable:

- Accession codes, unique identifiers, or web links for publicly available datasets
- A description of any restrictions on data availability
- For clinical datasets or third party data, please ensure that the statement adheres to our policy

The mass spectrometry proteomics data have been deposited to the ProteomeXchange Consortium via the PRIDE partner repository with the dataset identifiers PXD042858 (in vitro experiment) and PXD042905 (ECSIT experiment with cell extracts).
The cryo-EM maps have been deposited in the Electron Microscopy Data Bank (EMDB) under accession codes EMD-17659 (ACAD9-WT in complex with ECSIT-CTER); EMD-17660 (Cryo-EM structure of human ACAD9-S191A); and EMD-17661 (ACAD9 homodimer WT).
The atomic coordinates have been deposited in the Protein Data Bank (PDB) under accession codes PDB-8PHE (ACAD9-WT in complex with ECSIT-CTER) and PDB-8PHF (Cryo-EM structure of human ACAD9-S191A) (Supplementary Fig. 3, 4, 5 and Supplementary Table 1).
The source data underlying Figures 2A-I, 3F, 4C-F, 5C-E, 6A-G and Supplementary Figures 1A-C, 2A,B, 10A are provided as a Source Data file.

Research involving human participants, their data, or biological material

Policy information about studies with human participants or human data. See also policy information about sex, gender (identity/presentation), and sexual orientation and race, ethnicity and racism.

Reporting on sex and gender	N/A
Reporting on race, ethnicity, or other socially relevant groupings	N/A
Population characteristics	N/A
Recruitment	N/A
Ethics oversight	N/A

Note that full information on the approval of the study protocol must also be provided in the manuscript.

Field-specific reporting

Please select the one below that is the best fit for your research. If you are not sure, read the appropriate sections before making your selection.

☒ Life sciences ☐ Behavioural & social sciences ☐ Ecological, evolutionary & environmental sciences

For a reference copy of the document with all sections, see nature.com/documents/nr-reporting-summary-flat.pdf

Life sciences study design

All studies must disclose on these points even when the disclosure is negative.

Sample size	The sample size was dependent on the type of assay performed, as described in the Methods section, source data and figure legends.<br>For the cryo-EM studies, the numbers of micrographs collected and particles used are described in the methods section and in Supplementary Fig. 3 and Supplementary Table 1. The numbers of micrographs used were collected during 48hour sessions on a Thermofisher Titan Krios microscope operated with EPU. The micrographs selected enabled the determination of each structure to the resolution described in the paper.
Data exclusions	The numbers of particles retained for the final cryo-EM reconstructions are indicated in both the Methods section, and in Supplementary Fig. 3 and Supplementary Table 1.
Replication	The expression and purification of each of the proteins described in this study was conducted more than three times, showing similar results each time.<br>ITC, Mass Photometry, and SEC-MALLS, each experiment was conducted three times with similar results.<br>For the DLS measurements, at least three biological replicates including two to four technical replicates were measured for each sample with similar results.<br>Three to four replicates were conducted for the Acyl-CoA dehydrogenase (ACAD) activity assay with similar results.<br>For the CI activity assay, each assay was carried out with three independent experiments and results were presented as a mean average with the standard deviation (s.d.).<br>The human Aβ1-42 content in isolated mitochondria from WT and APP cells was measured three independent experiments with similar results.<br>The phosphorylation assays, both in vitro and using cell extracts, were repeated twice with similar results.<br>Mass spectrometry in-gel digestion experiments were conducted

	Eight cryo-EM grids were prepared and screened for the ACAD9-ECSIT-CTER complex, eight for ACAD9-WT and eight for ACAD9-S191A. Each batch of grid preparation showed similar particle distribution and overall character. The grids with the highest ice quality were selected for data collection.
Randomization	For the cryo-EM analysis, refinement was conducted according to the gold-standard refinement protocol, involving the random assignment of particles to two independent half-datasets.
Blinding	The investigators were blinded to group allocations.

Reporting for specific materials, systems and methods

We require information from authors about some types of materials, experimental systems and methods used in many studies. Here, indicate whether each material, system or method listed is relevant to your study. If you are not sure if a list item applies to your research, read the appropriate section before selecting a response.

Materials & experimental systems

n/a	Involved in the study
☐	☒ Antibodies
☐	☒ Eukaryotic cell lines
☒	☐ Palaeontology and archaeology
☒	☐ Animals and other organisms
☒	☐ Clinical data
☒	☐ Dual use research of concern
☒	☐ Plants

Methods

n/a	Involved in the study
☒	☐ ChIP-seq
☒	☐ Flow cytometry
☒	☐ MRI-based neuroimaging

Antibodies

Antibodies used	Complex I specific antibody pre-coated in a microplate wells (Abcam ab109721) to purify CI from cell extracts and measure CI activity. Amyloid beta 42 Human ELISA containing an Amyloid beta 42 antibody pre-coated in a microplate wells and a biotinylated detection antibody (ThermoFisher khb3544).
Validation	Validation of the antibodies listed above can be obtained by typing the references on the manufacturer websites: 1. Complex I antibody: https://www.abcam.com/products/assay-kits/complex-i-enzyme-activity-microplate-assay-kit-colorimetric-ab109721.html; 2. Amyloid beta 42 antibody: https://www.thermofisher.com/elisa/product/Amyloid-beta-42-Human-ELISA-Kit-Ultrasensitive/KHB3544.

Eukaryotic cell lines

Policy information about cell lines and Sex and Gender in Research

Cell line source(s)	Human neuroglioma H4 cells, both wild-type (WT) and stably transfected with human amyloid precursor protein (APP) carrying the AD-related Swedish mutation (KM670/671NL), derived from the ATCC catalogue (https://www.atcc.org/products/htb-148).
Authentication	The cell lines were not authenticated for this study.
Mycoplasma contamination	Cell lines tested negative for mycoplasma contamination.
Commonly misidentified lines (See ICLAC register)	No commonly misidentified lines were used in this study.
